# Response of breast cancer cells to IFNα-2b in 2D and 3D cell cultures

**DOI:** 10.3906/biy-1808-36

**Published:** 2019-02-07

**Authors:** Tetiana HERHELIUK, Olena PEREPELYTSINA, Andrij UGNIVENKO, Lyudmila OSTAPCHENKO, Mikhailo SYDORENKO

**Affiliations:** 1 Department of Biotechnical Problems of Diagnostics, Institute for Problems of Cryobiology and Cryomedicine, National Academy of Science of Ukraine , Kyiv , Ukraine; 2 Educational and Scientific Centre “Institute of Biology & Medicine” , Kyiv , Ukraine

**Keywords:** Breast cancer, MCF-7, 2D cell culture, multicellular tumor spheroids, interferon alfa

## Abstract

The effect of IFNα-2b on the migration, proliferation, and expression of epithelial and mesenchymal markers of MCF-7 tumor adenocarcinoma cells in 2D and 3D cell cultures was examined. A significant cytostatic effect of IFNα-2b on the tumor population was detected. It was found that changes in the expression of epithelial (CKs and EpCAM) and mesenchymal markers were caused by changing the growth type of the tumor population. IFNα-2b inhibited migration of tumor cells to the suspension fraction and promoted an increase in expression of CK and EpCAM in 2D and 3D cell cultures, but only in the 3D culture was expression of vimentin increased. IFNα-2b caused an increase in CK and EpCAM expression by 50.5% and 47.8%, respectively, compared with the control in the 2D cell culture. In the 3D cell culture this increase was 33% and 34%, respectively, compared with the control. IFNα-2b stimulated the differentiation and inhibited the migrational ability of tumor cells in the early stages of breast cancer development.

## 1. Introduction

It is generally accepted that cancer cells arise from healthy cells that have undergone genetic or epigenetic changes (Erenler and Geçkil, 2014) . During tumor development, the tumor microenvironment, which contains stromal and immune cells as well as cytokines produced by these cells, plays a determining role (Hanahan and Coussens, 2012) . Previous studies have shown that different populations of immune cells and the molecules they produce are important in the progression of tumors (Zarour, 2016) . It is also well known that the growth of most malignant neoplasms is accompanied by certain impairment of the immune response (Kadegidze et al., 2013) . Inflammatory reactions play an important role in all stages of the development of the tumor, such as the formation of micrometastases, the acquisition of malignant phenotypes, and intravascular spread. These data served as the basis for the widespread use of oncology therapeutic agents that can restore the functions of the immune system. Interferons (IFNs) are one of the most important regulators of the human immune system. They are a group of cytokines that are able to exert direct and indirect effects on tumor cells. Thus, interferons have antiproliferative, antiviral, and immunomodulating properties (Hsu et al., 2016) . Due to this, IFNα-2b is used as an antiproliferative agent during monotherapy or combination therapy with other antitumor drugs (Ningrum, 2014) . IFNα-2b is of obvious importance in anticancer therapy because it affects all aspects of cellular and humoral immunity, regulation of hematopoiesis, and synthesis and production of various cytokines, causing an inhibitory effect on malignant cells.

Shift to the mesenchymal phenotype causes an increase in the migratory capacity of tumor cells (Lamouillle et al., 2014) . Epithelial–mesenchymal transition (EMT) can also be caused by local inflammation. During this process, tumor cells partially or completely lose their epithelial characteristics (EpCAM and CK) and acquire mesenchymal phenotypes (vimentin), which increase tumor cell plasticity, so as to easily escape from the primary tumor into blood (Kim et al., 2014) . Few researchers have addressed the question of searching for factors that can inhibit the transition of the cell population from the epithelial to the mesenchymal phenotype (SuarezCarmona et al., 2017) . The past decade has seen renewed importance placed on interferon alfa (IFNα-2b) as a factor capable of modifying EMT of the tumor population during the development of the tumor process. Several authors have shown that long-term therapy of human cancer cells using this cytokine leads to changes in epithelial and mesenchymal markers indicating suppression of the EMT program (Semesiuk et al., 2011) . Since EMT is associated with processes for the migration of tumor cells and the formation of micrometastases, it is extremely important to study the effect of IFNα-2b on this process.

Cancer cell lines are widely used as models for studying the mechanisms of cancer development and the study of the effectiveness of antitumor agents. The environment conditions in monolayer culture (2D) in vitro differ significantly from in vivo conditions, since the tumor population is fairly heterogeneous and consists of cells at different stages of development and differentiation. In addition, in natural conditions, cells in the tumor interact with adjacent cells and the extracellular matrix, and also have different access to nutrients and oxygen (Vidyasekar et al., 2016) . Oeftn these diefrences are the cause of the ineefctiveness of antitumor therapy, which showed promising results in preclinical studies in 2D cell growth conditions in vitro. An alternative model for the study of tumor cell susceptibility to antitumor agents is multilayered spherical 3D cultures or multicellular tumor spheroids (MCTSs) (Friedrich, 2007) .

Cells in 3D culture actively interact with each other, the extracellular matrix, and the microenvironment. Such interactions effect cell proliferation, differentiation, and morphology; gene expression; and protein synthesis. The structure of 3D tumor aggregates is similar to that of a tumor at an early avascular stage of development or to micrometastases. In addition, MCTSs consist of cells that are at different stages of their development and under different inuflences (proliferative, restless, apoptotic, hypoxic, and necrotic cells) (Kim, 2005) . Due to their structure, MCTSs are important for testing the therapeutic effect of antitumor drugs, as well as for assessing the invasive capacity of transformed cells. The aim of the present study was to evaluate the effect of IFNα-2b on the migration, proliferation, and expression of epithelial and mesenchymal markers of MCF-7 tumor adenocarcinoma cells in 2D and 3D cell cultures.

## 2. Materials and methods

### 2.1. Cultivation of the monolayer culture

MCF-7 cells were obtained from the Cell Line Bank of the Kavetskii Institute of Experimental Pathology, Oncology, and Radiobiology, National Academy of Sciences of Ukraine, and maintained in DMEM (Sigma, St. Louis, MO, USA) with 40 mg/mL gentamicin (Biopharma, Ukraine), 2 mM L-glutamine (Sigma), and 10% fetal bovine serum (FBS) (Sigma) in a 5% CO2 incubator that was maintained at 37 °C. Initial cell density was 2 × 104 cells/cm2. The cells were used in the experiments after 2 days of incubation.

### 2.2. Cultivation of the spheroid culture

MCTS generation started with cell removal from the substrate using 0.25% trypsin-EDTA (Sigma) and transfer to DMEM with 2% carboxymethyl cellulose (BioRad, Hercules, CA, USA) to the final concentration of 5 × 105 cells/mL. The cells were subsequently incubated in an orbital shaker (PSU-10i, Biosan, Riga, Latvia) at 80 rpm for 3–5 h. Half of the culture medium was changed every 3 days. The spheroid cultures were maintained for 7 days under these conditions.

### 2.3. Cell proliferation and viability

The effect of IFNα-2b on the proliferative activity and adhesion properties of the tumor population was analyzed in MCF-7 cells incubated with IFNα-2b (Laferobion, Biopharma, Kyiv, Ukraine) at concentrations of 103, 104, and 105 U/mL. The dye exclusion test was used to determine the number of viable cells present in a cell suspension and adhesion after 24 h. It is based on the principle that live cells possess intact cell membranes that exclude certain dyes, whereas dead cells do not. We mixed 20 μL of 0.4% Trypan blue solution with 20 μL of the cell suspension and counted the number of living cells using a hemocytometer. Cells incubated in standard DMEM under standard conditions were used as controls. Cell viability was evaluated by MTT assay (Mossman, 1983) . MCF-7 cells were incubated at 1 × 104 cells/well in a 96well plate and grown overnight. The cells were treated with various concentration of IFNα-2b, as indicated, for 24 h and 48 h. At the end of these periods, 100 μL of MTT (3-(4,5-dimethylthiazol-2-yl)-2,5-diphenyltetrazolium bromide) at a dose of 5 mg/mL (Sigma) was added to each well, followed by further incubation for 4 h at 37 °C. The supernatant was removed and then 400 μL of 0.1% dimethyl sulfoxide was added to all wells to dissolve the purple formazan crystals. The dissolved crystals were transferred to a 96-well plate for reading and measured with a microplate spectrophotometer (eThrmo/LabSystems 352 Multiskan MS Microplate Reader) at a wavelength of 540 nm.

### 2.4. Morphometric assay of MCTSs

The cell aggregates were photographed after 7 days of cultivation and their sizes were measured in order to characterize the parameters of MCTSs in the 3D culture. Stemi 2000 software (Zeiss, Germany) was used for image processing, and the Bjerkvig formula (V = 0.4 × a × b2, a – largest size of spheroid, b – smallest size of spheroid) was used to calculate aggregate volume (Bjerkvig, 1991) .

### 2.5. Immunohistochemistry

The expression of markers under the influence of IFNα2b was assessed in 2D cultures maintained on glass coverslips in 6-well plates at a density of 2 × 104 cells/ cm2. Receptor expression was analyzed after 2 days of incubation. The MCTSs were embedded in parafin blocks. The markers were visualized immunohistochemically with the following monoclonal primary antibodies: CK (clone AE1/AE3, IS053, Dako, Carpinteria, CA, USA), vim (Clone V9, IS630, Dako), and EpCAM (HPA026761, Sigma, Stockholm, Sweden). Histological samples of the cells were photographed for comparing the morphological characteristics and expression of the receptors in both monolayer and spheroidal cell cultures. In the cell cultures under study, the number of immunopositive/immunonegative cells was determined. The number of immunopositive cells was counted in 10 randomly selected microscope fields for each sample using a standard measuring scale (object - micrometer) at the same magnitude and calculated as a percentage of the total number of cells taken at 100%. At least 1000 cells were counted.

### 2.6. Cell cycle analysis

Cell cycle analysis was performed using propidium iodide staining, as described previously (Zhao et al., 2015) . Distribution of the MCF-7 cell population by cell cycle was determined by flow cytometry (Becton Dickinson) at λir = 488 nm and λem = 585/40 nm.

### 2.7. Statistical analysis

One-way analysis of variance and Student’s t-test used for statistical processing of the data were implemented in the software package Statistica 8. The significance threshold was set at P ≤ 0.05. The results are presented as mean values and standard error values (M ± SE).

## 3. Results

**Figure 1:**
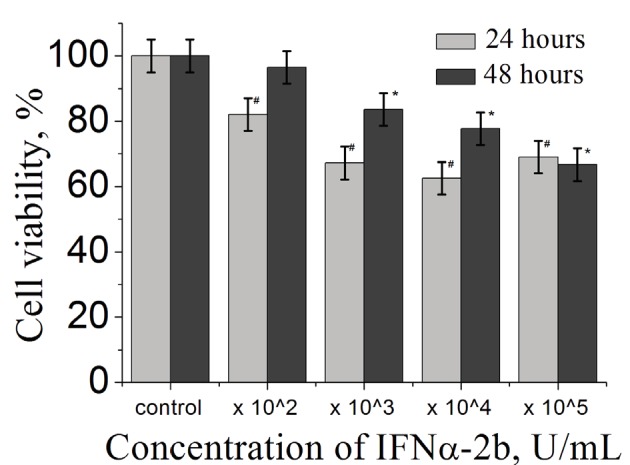
Survival of MCF-7 cells in the monolayer culture with various concentrations of IFNα-2b (102, 103, 104, 105 U/mL). Cell viability (%) was determined by the MTT assay on the first and second days of cultivation, *, # - P ≤ 0.05.

In the first stage of tumor formation, we evaluated the survival and proliferative activity of tumor cells when they were incubated with IFNα-2b by MTT analysis in a cell monolayer culture at different concentrations of IFNα-2b after 24 and 48 h of cultivation. Survival of tumor cells in control specimens under standard culture conditions was taken as 100% (Figure 1). Figure 1 shows the cytotoxic effect of IFNα-2b on the first and second days of cultivation. Thus, the reduction in survival of tumor cells was maximal at the IFNα-2b concentration of 104 U/mL on the second day of cultivation. It was also found that survival of tumor cells incubated in the presence of IFNα2b was lower by 47% compared with control samples. It is interesting that cell viability increases in a concentration of 105 U/mL and the numbers of living cells are more than 103 and 104 U/mL after 24 h of incubation. IFN-α has a pleiotropic effect, because it stimulates antitumor immune response and also directly affects the proliferation and survival of tumor cells. However, as it turned out, these are not all the positive effects of this cytokine. Ma et al. (2017) demonstrated that IFNα can upregulate cancer stem cell (CSC) markers and can activate dormant CSCs. They found that IFN-α can enrich tumors by CSCs while at the same time killing the bulk of tumor cells. That is why we think that in the present time of cultivation IFNα-2b at concentration 105 U/mL enhanced the number of living cells, because the population of CSCs was differentiated into tumor cells. However, after 24 h (48 h of cultivation) IFNα-2b inhibited the proliferation of tumor cells because of its cytotoxicity effect.

**Figure 2:**
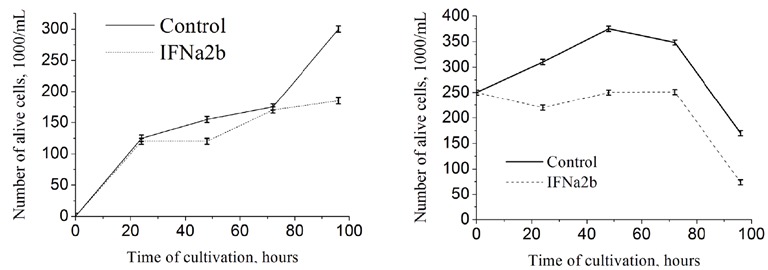
Survival of MCF-7 cells in the presence of IFNα-2b, suspension (A), and adhesive (B) fraction, * - P ≤ 0.05. Alive cells in the suspension and adhesion fractions were counted after 24 h according to a conventional procedure that involved Trypan blue staining.

The ability of tumor cells for substrate-independent growth underlies the processes of migration and metastasis. The data obtained in the first stage give an understanding of the general survival of the cell population of a monolayer culture. However, an indicator of the migration activity of tumor cells is the change in adhesion ability. In order to evaluate the adhesion potential of tumor cells, we analyzed how the composition of the adhesion and suspension fraction of monolayer culture changes over 4 days of cultivation. MCF-7 cells were incubated with IFNα-2b at a concentration of 2 × 104 U/mL. Every 24 h living cells were counted in the suspension and adhesive fractions (Figures 2A and 2B). The present study showed that IFNα2b had a cytostatic effect on the tumor population, keeping the number of living cells in both the suspension and the adhesion fraction at a level lower than the control. It was demonstrated that the number of living cells cultured with IFNα-2b in the adhesion fraction showed a cytotoxic effect of cytokine on days 3–4 of cultivation (Figure 2A), since during this period there was a decrease in the number of cells by 29% and 59%, respectively, in comparison with the control. In the suspension fraction, on day 4 of cultivation (Figure 2B), the number of cells was less than 40% compared to the control. At about 72 h, the number of living cells decreases in both the control and IFNα-2b groups in the adhesion fraction with the same slope, at almost the same time. However, in Figure 2A the number of living cells in the suspension fraction increases after 72 h of cultivation. The fact is that part of the adherent cells go into suspension. At the same time, IFNα-2b inhibits the transition from the adhesion to the suspension fraction, compared with the control. We suppose that the migration capacity of tumor cells is lower under the influence of IFNα-2b.

MCTS is a model for the primary stage of development of breast cancer, and so it is very important to evaluate the effect of IFNα-2b precisely at this stage of disease development. It is known that MCTSs in vitro are formed as a result of migration and aggregation of cells (Vinci et al., 2013) . The analysis of the formation of MCTSs, based on the average size of aggregates in culture, indicates a decrease in the size of tumor spheroids that have been incubated with IFNα-2b. The dose-dependent decrease in spheroids was greater when IFNα-2b was added: 3.67 (102 U/mL) and 1.68 times (104 U/mL), respectively (Table 1).

**Table 1:** Volume of multicellular tumor spheroids of the MCF-7 cell line after incubation with IFNα-2b. *Values ± standard error after 5 repetitions are given, Р ≤ 0.05.

Concentration of IFNα-2b, U/mL	Volume, mm*10^-3^
0	0.99 ± 0.05
10^2^	0.27 ± 0.01
10^3^	0.65 ± 0.03
10^4^	0.46 ± 0.02

Since interferons can act as cell cycle regulators, we analyzed induction of apoptosis or cell cycle stop under the influence of IFNα-2b in MCF-7 cells at a concentration of 104 U/mL. Analysis by flow cytometry showed that IFNα2b causes cellular accumulation in the G0/G1 phase (Table 2), which may indicate induction of apoptosis and cell cycle arrest by this cytokine. The decreasing cell number in the synthetic phase of the cell cycle possibly caused inhibition of MAP kinase activity, a key regulator of Cdk at different stages of the cell cycle.

**Table 2:** Spreading of MCF-7 cell population on phases of the cell cycle in the 2D cell culture under the influence of IFNα-2b (10^4^ U/mL).

Phases of cell cycle	Control of MCF-7 cells	MCF-7 cells + IFNα-2b
G0/G1	41%	53%
G2/M	24%	29%
S	35%	18%

The next step in the study was to analyze the expression of epithelial (CK and EpCAM) and mesenchymal (vim) markers under the influence of IFNα-2b. Thus, in the monolayer culture of tumor cells in the presence of cytokine (104 U/mL), the expression of CK and EpCAM increased by 50.5% and 47.8%, respectively, as compared with the control, but expression of vimentin was not observed in this culture (Table 3).

**Table 3:** Expression of tumor markers in the presence of IFNα- 2b, Р ≤ 0.05.

	CK, %	EpCAM, %	Vim, %
			0
			0
			5 ± 0.3
			7 ± 0.4

In the spheroid culture of tumor cells, the expression of CK and EpCAM in the presence of interferon alfa increased by 33% and 34%, respectively, compared with the control. However, the increase in the expression of vimentin was slightly different from that of the control (by 2%). Thus, interferon alfa enhances the expression of the epithelial markers EpCAM and cytokeratins both in the monolayer (Figure 3A) and in the cells of MCF-7 spheroids (Figure 3B). Since there was an increase in the expression of epithelial markers and there was no increase in the expression of vimentin, it can be assumed that interferon alfa did not lead to the EMT phenomenon, since tumor cells, in contrast, acquire distinct signs of differentiation.

**Figure 3:**
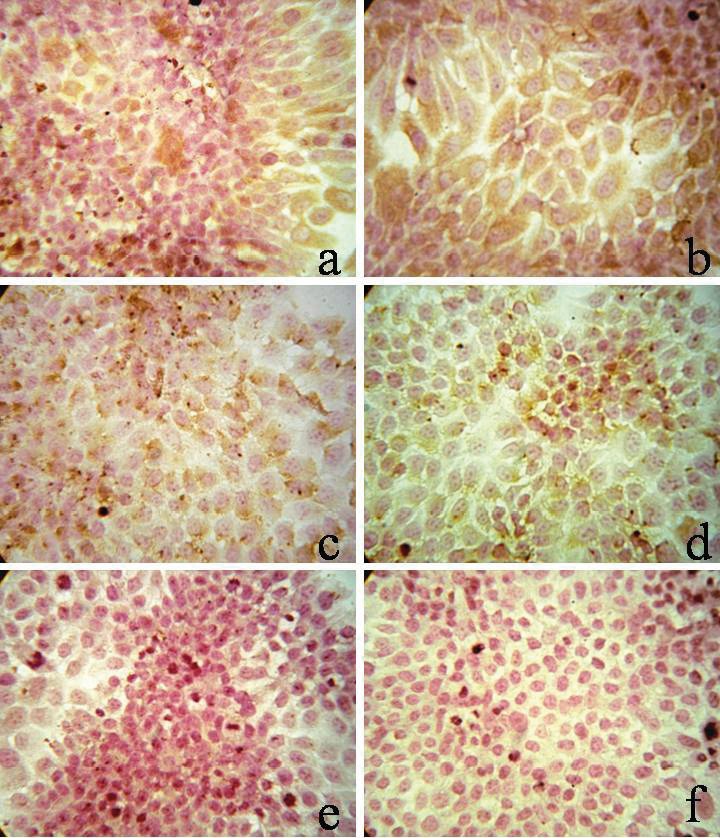
Expression of tumor markers in 2D (A) and 3D (B) MCF-7 cell cultures by IFNα- 2b (104 U/mL), microphotography, hematoxylin/eosin, 400× magnification: a) CK expression, MCF-7 control, b) Expression of CK, MCF-7 + IFNα-2b, c) EpCAM expression, MCF-7 control, d) EpCAM, MCF-7 + IFNα-2b expression, e) Vim expression, MCF-7 control, f) Vim expression, MCF-7 + IFNα-2b.

**Figure 3(continued):**
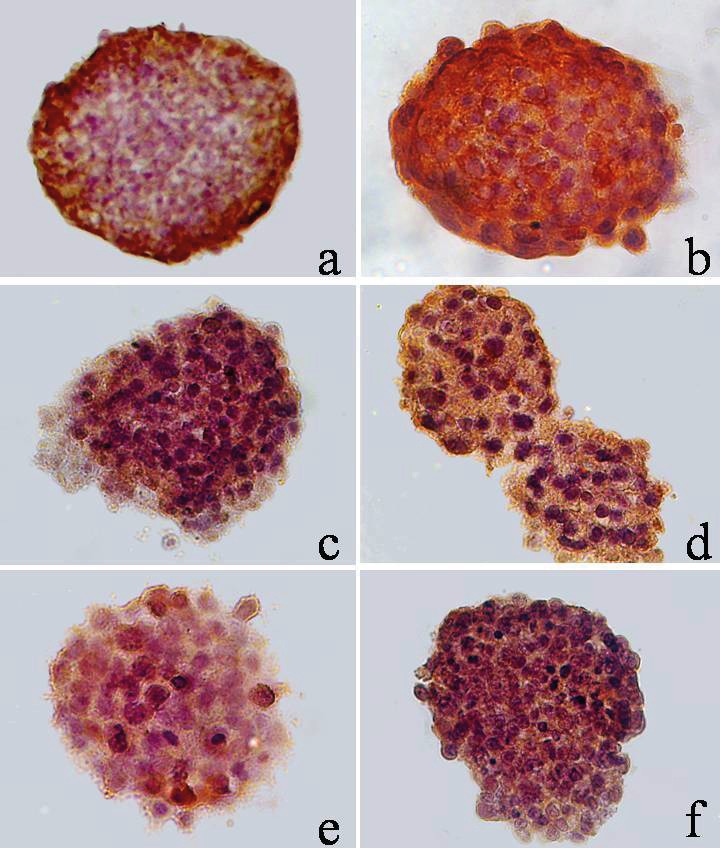
Expression of tumor markers in 2D (A) and 3D (B) MCF-7 cell cultures by IFNα- 2b (104 U/mL), microphotography, hematoxylin/eosin, 400× magnification: a) CK expression, MCF-7 control, b) Expression of CK, MCF-7 + IFNα-2b, c) EpCAM expression, MCF-7 control, d) EpCAM, MCF-7 + IFNα-2b expression, e) Vim expression, MCF-7 control, f) Vim expression, MCF-7 + IFNα-2b.

## 4. Discussion

It was shown that IFNα-2b inhibited the proliferation and migration ability of MCF-7 cells. IFNα-2b also enhanced expression of epithelial markers by MCF-7 cells in 2D and 3D cell cultures. Type I interferons (IFNα, IFNβ, IFNω) are involved in the processes of anticancer defense and cell differentiation, as well as being capable of increasing the sensitivity of tumor cells to chemotherapy and radiation therapy (Morak et al., 2011) . These substances act through the formation of a receptor complex after binding to a receptor, which consists of two subunits of IFNAR-1 and IFNAR-2. The IFNAR-2 subunit is represented by a soluble form and can act as a dominant negative regulator of free interferons; the IFNAR-1 subunit is a shorter form in which there are no cytoplasmic domains. IFNAR-2 contains the whole cytoplasmic domain and, together with IFNAR-1, is a functional IFNR receptor that is able to bind IFN and induce JAK-STAT signaling (Bekisz et al., 2004) . As is known from the literature, IFNα stimulates phagocytosis of macrophages and neutral granulocytes, and activates the production of reactive oxygen species in them, thereby increasing the cytotoxicity of cells, increasing the synthesis of interleukin 1 (IL-1) phagocytes and tumor necrosis factor (TNF-α), causing expression on HLA Class I membranes required for the recognition of target cells, including tumorous, cytotoxic lymphocytes, and for the functioning of T-suppressors (Lisyany et al., 2004) .

We suggest that IFNα-2b inhibits proliferation of breast cancer cells in the system of 2D and 3D cell growth. Such an antiproliferative effect can be achieved by stopping a cell cycle, apoptosis, or differentiation. In the body, its action may be mediated by the activation of immune cells (T cells and natural killers), inhibition of vascularization (angiogenesis), and induction of cytokines that control various processes in the cell, such as proliferation, differentiation, survival, and apoptosis (Bekisz et al., 2010) . Compared to monolayer culture, MCTS cells are less susceptible to IFNα-2b, due to the heterogeneity of cell populations in this model. MCTS outer layers consist of actively proliferating cells and the core of the unit is enriched with cells in rest or hypoxia, because they receive less oxygen, growth factors, and nutrients. It is also known that MCTSs contain about 1% of stem cell tumors capable of resuming a tumor cell population. In addition, 2D and 3D cultures of cancer cells differ in the level of gene expression, the intensity of proliferation, and the ability of cells to migrate and metastasize (Gurski et al., 2010) .

One of the stages of the study of tumor cell susceptibility to IFNα-2b was the analysis of the cell division cycle, since its violation leads to uncontrolled growth of the tumor population. Normally, cells in an organism are in one of three states: in a loop, in a resting state, with the preservation of the ability to return to the cycle, and in the stage of final differentiation, when the ability to divide is lost. Of course, tumors can form only those cells that can actively divide. The spread of tumor cells and the growth of the tumor is a complex process that involves many successive stages, in which positive and negative regulators of the cell cycle are involved. One of the crucial stages of the cell cycle in mammalian cells is the G1 phase, during which the cell “decides” to stop or continue a new cell division cycle. Cell cycle regulation and proliferation may be carried out, primarily, by extracellular signals, in particular cytokines (interleukins and interferons) (Grana and Reddy, 1995). We have shown that IFNα-2b stops the cell cycle of cancer cells in the G0/G1 phase, causing apoptosis. There are studies that confirm this finding (Ossina et al., 1997; Chawla-Sarkar et al., 2001; Thyrell et al., 2002) . A team of scientists has shown that IFNα can stop the cell cycle in the G1 phase by activating JAKSTAT signaling, and also suppresses proliferation of tumor cells. In other studies, the value of IFNα as a regulator of expression of cyclin-dependent kinase inhibitors (Cdks) is described; furthermore, p21 induction is a major event in the regulation of the cellular cycle mediated by IFNα (Szeps et al., 2003) . When cells of epithelial origin, in this case tumor cells of the mammary gland, become signs of the mesenchymal phenotype, they acquire the ability to suppress the antitumor protective forces of the organism, migrate to the body, become incapable of apoptosis and insensitive to the action of antitumor drugs, and act as a reservoir that replenishes and expands a population of tumor cells (Marcucci et al., 2016) . Tumor cells that undergo EMT are actively proliferating and self-renewed, resulting in an increase in the number of heterogeneous populations. As a result, the pool of migrating cells is replenished, increasing cellular mobility, which results in the formation of metastatic colonies in remote locations (Christofori and Bill, 2015) . That is why the ability of tumor cells to express markers characteristic of mesenchymal and epithelial gene cells has been analyzed in this work. It has been shown that IFNα-2b does not effect the enhancement of the features that are characteristic of cells of the mesenchymal phenotype, but it promotes cell differentiation and expression of the markers of the epithelial phenotype. Future work should concentrate on exploring the possibility of combining IFNα-2b with agents aimed at destroying or differentiating the cancer stem cell population in targeted antitumor therapy.

Thus, we can conclude that IFNα-2b demonstrated cytotoxic properties and capacity to reduce the intensity of MCTS formation migration activity of breast cancer cells. MCTSs are less sensitive to IFNα-2b due to different cell populations in its composition. Changing the growth of the tumor population causes changes in the expression of epithelial and mesenchymal markers. Thus, the percentage of epithelial markers in MCTSs is less than in monolayer cells; however, spheroid cells begin expressing a mesenchymal marker: vimentin. In the 2D culture, tumor cell culture of IFNα-2b promoted increased expression of CK and EpCAM by 50.5% and 47.8%, respectively, as compared to the control. In the 3D cell culture, this increase was 33% and 34%, respectively, compared to the control. In MCTSs only the outer cell layers expressed these proteins, unlike adhesive cells, which all showed equal expression. As there was an increase in the expression of epithelial markers and the expression of vimentin was not significantly different from that of the control, it can be concluded that IFNα-2b positively affects the acquisition of signs of differentiation, as well as causing a decrease in the migration capacity of tumor cells in the early stage of breast cancer development. Thereby, MCTSs may be considered as a model for studying the EMT process in vitro and as a test system with IFNα-2b for immunomodulatory anticancer therapy.
